# Neurological symptoms and disorders following electrical injury: A register-based matched cohort study

**DOI:** 10.1371/journal.pone.0264857

**Published:** 2022-03-02

**Authors:** Kent J. Nielsen, Ole Carstensen, Anette Kærgaard, Jesper Medom Vestergaard, Karin Biering

**Affiliations:** Department of Occupational Medicine—University Research Clinic, Danish Ramazzini Centre, Goedstrup Hospital, Herning, Denmark; University of Gothenburg: Goteborgs Universitet, SWEDEN

## Abstract

**Introduction:**

Electric shocks may have neurological consequences for the victims. Although the literature on the neurological consequences of electric shocks is limited by retrospective designs, case studies and studies of selected patient groups, previous research provides some evidence of a link between electric shocks, and diseases and symptoms of the central nervous system (CNS)(e.g. epilepsy, migraine and vertigo) and the peripheral nervous system (PNS)(e.g. loss of sensation, neuropathy and muscle weakness). This study aims to employ a register-based, matched cohort study, to investigate whether individuals demonstrate a greater risk of neurological diseases and symptoms of the CNS or PNS in the years following an electrical injury.

**Materials and methods:**

We identified 14,112 electrical injuries over a period of 19 years in two Danish registries, and matched these with three different groups of persons in a prospective matched cohort study: (1) patients with dislocation/sprain injuries, (2) patients with eye injuries and (3) persons employed in the same occupation. Year of injury, sex and age were used as matching variables. The outcomes we identified comprised neurological disorders and central or peripheral nervous system symptoms that covered a range of diagnoses in the Danish National Patient Register. The associations were analysed using conditional logistic regression for a range of time periods (six months to five years) and conditional Cox regression for analyses of the complete follow-up period (up to 20 years).

**Results:**

For victims of electric shock, the CNS sequelae we identified included an increased risk of *epilepsy*, *convulsions*, *abnormal involuntary movements*, *headache*, *migraine* and *vertigo*. We also identified an uncertain, increased risk of *spinal muscular atrophy* and *dystonia*, whereas we identified no increased risk of *Parkinson’s disease*, *essential tremor*, *multiple sclerosis* or *other degenerative diseases of the nervous system*. For victims of electric shock, the PNS sequelae we identified included an increased risk of *disturbances of skin sensation*, *mononeuropathy* in the arm or leg and *nerve root and plexus disorders*. We also identified an uncertain, increased risk of *facial nerve disorders*, *other mononeuropathy*, and *polyneuropathy*.

**Conclusion:**

Our results confirm that electrical injuries increase the risk of several neurological diseases and symptoms of the CNS or PNS in the years following the injury. Most often the diseases and symptoms are diagnosed within the first six months of the injury, but delayed onset of up to 5 years cannot be ruled out for some symptoms and diagnoses. Some of the conditions were rare in our population, which limited our ability to identify associations, and this warrants cautious interpretation. Therefore, further studies are needed to confirm our findings, as are studies that examine the mechanisms underlying these associations.

## Introduction

Electric shocks may have both serious immediate consequences, such as burns or cardiac arrest caused by the current, and serious secondary physical injuries, for example, those caused by falling or being thrown back by the shock. These serious, immediate consequences may be the most notable and well-known, but electric shocks may also have both immediate and delayed neurological consequences [1, 2]. This is the focus of this paper, which examines the risk of developing conditions and symptoms of the central (CNS) or peripheral nervous system (PNS) in the years following an electrical injury.

Previous research has yielded mixed evidence of a link between electric shocks and diseases and symptoms of the CNS. For instance, a review of the literature based primarily on case studies and patient groups from burn units reported an association of electric shocks and an increased risk of epilepsy, Parkinson’s disease and Amyotrophic Lateral Sclerosis (ALS), first manifesting a significant time after the electric shock [2]. However, a case-control study that used a combination of Job Exposure Matrices and retrospective, self-reported exposure did not confirm any increased risk of Parkinson’s disease [3]. Furthermore, a Danish register-based study that followed all persons who reported an electrical injury to the Danish Safety Technology Authority between 1968 and 2008 found no increased risk of ALS, Multiple sclerosis, Parkinson’s or Alzheimer’s disease during follow-up, whereas an increased risk was found of migraine, vertigo, and epilepsy, based on Standardised Hospitalisation rates [4]. Several other symptoms related to the CNS, such as headache, general fatigue and tremor, have been reported following electric shocks [1, 5–11].

Research has also indicated a link between electric shocks and conditions and symptoms of the PNS, although the aetiology behind this possible association is debated [2, 6]. The manifestations may be chronic PNS symptoms following the shock, such as neuropathic pain, loss of sensation, paraesthesia and muscle weakness [8, 12]. A Danish cohort study found an increased risk of peripheral nervous disease following electric shock, compared to the incidence in the general population [4], and in a Canadian multi-centre study of previously hospitalised electrical injury patients, 9% reported muscle weakness, 6% extremity tingling and 9% numbness of the limbs at the one-year follow-up [10]. Furthermore, a retrospective study of 311 electrical workers suffering from electrical injuries found that 20 persons reported neurological sequelae, of which peripheral nervous disturbance comprised 90% [13]. Studies from burn units report a larger proportion of peripheral neuropathy following electrical burns, compared to non-electrical burns, and that high voltage injuries accounted for most of them [14, 15]. Several papers divide these injuries into low- and high-voltage injuries, as a measure of severity. However, this is probably too simplistic, as many other factors, such as duration, type of current, resistance of the tissue in the current’s path and the humidity of surroundings are crucial for the consequences of the shock [1, 6].

Generally, the literature on the neurological consequences of electric shocks is limited by retrospective designs, case studies and studies of selected patient groups, which are prone to recall and/or selection bias. Furthermore, there is a lack of comparison groups, so casuistic and descriptive cohort studies dominate the field, which is also reflected in reviews [1, 2].

This study aims to employ a register-based, matched cohort study, to investigate whether individuals demonstrate a greater risk of neurological diseases and symptoms of the central or PNSs in the years following an electrical injury.

## Materials and methods

### Materials

This study was a matched cohort study based on injuries registered in two population-based registers: The Danish National Patient Register (DNPR) and the register of work injuries reported to The Danish Working Environment Authority (DWEA). Furthermore, it included data from other population-based registers in Statistics Denmark, described in detail in the following sections.

The DNPR covers all hospital contacts in Denmark, including information regarding diagnoses and procedures carried out during in- and outpatient, and casualty department visits [16, 17]. Mandatory registration of accidents in the DNPR began in 2000. Before that, the diagnosis code DT754 was sometimes used to register accidents, but not necessarily, if the main problem following the accident was something else, such as a burn or unconsciousness. The DNPR began to use ICD-10 codes in 1994, and we had data that went to the end of 2016.

The DWEA register lists work injuries reported by employers, employees, unions and healthcare workers. In Denmark, it is mandatory for employers to report any work injury that results in sick leave lasting at least the day following the day of the injury. The DWEA register exists to support compensation claims, but the reporting system is also designed to provide an overview of work injuries [18]. Statistics Denmark provided information on injuries from the DWEA register from 2005 to 2017.

The period studied covered registered Danish electrical injuries from 1994 to 2016, and we included electrical injuries from 1996 to 2014 in our analysis, to allow for at least two years prior to the injury, with no evidence of the sequelae of interest, and at least two years following the injury for the sequelae to develop.

Statistics Denmark is the central Danish registries and statistics authority. The range of topics covered includes the population register, which includes movement within and to/from Denmark, and nationality [19, 20]. Statistics Denmark also maintains employment and industry registers, based on the registers of employers/companies and taxpayers in Denmark [21], and the register of deaths [22]. Injury records from the DNPR and the DWEA were linked to Statistics Denmark by the unique personal identification numbers (CPR number) and injury date/year. Each Danish citizen and registered migrant worker has a unique CPR number that links each person to demographic and work-related registries [23]. The CPR number is encrypted for researchers, so individuals could not be identified.

Participants’ occupation was determined from the register-based labour-force statistics (RAS) register at Statistics Denmark by means of DISCO codes. DISCO is the official Danish version of International Standard Classification of Occupations (ISCO), prepared by the International Labour Organisation (ILO) [21]. Current working status was also derived from the RAS register, to define persons from the working population (employed, self-employed and assisting spouses, in contrast to unemployed, retired, studying or otherwise not working)

### Methods

#### Participants

The DNPR enabled us to identify patients with electrical injury (ICD10) diagnoses (DT754, EUHA10 and EUYZ203), related to contact with a hospital. The DT754 code was used throughout the period studied, whereas the two accident codes (EU*) have been used only since 2000, when a separate injury register was established and subsequently included in the DNPR. Both hospitalisations and outpatient visits were included.

In the DWEA register, persons with electrical injuries were identified by information regarding cause of injury. Two different codes defined exposure: ‘Acute/short exposure to welding arc or electrical arc’ and ‘Acute/short exposure to electricity or reception of electric charge in the body’.

If an injury was registered in both registers (+/− 7 days), only the first registration was used, regardless of the register in which it appeared.

#### Other variables

Sex, age and occupation at time of being matched for this study were derived from Statistics Denmark, as was the date of emigration or death, if applicable. We registered whether the patient was part of the workforce at the time of the injury. If the injury was identified in the DNPR, it was not necessarily work-related, whereas all injuries in the DWEA register were occupational injuries.

For sensitivity analyses we calculated the total length of hospitalisation, including time in the casualty department, and derived the diagnoses of concussion (S06.0) and diabetes (E10-E14) from the DNPR. Not all injuries from the DWEA register could be assigned a length of hospitalisation, if we could not identify any hospital contact at the time of the injury (+/− 7 days).

#### Matching

Each patient was matched in three different ways with persons from the same data source (DNPR or DWEA).

*Match 1 –dislocation/sprain*. Electrical injury patients were matched with up to ten other patients with a dislocation/sprain (DS93 in the DNPR and ‘sprains’ in the DWEA register)

*Match 2 –eye injury*. Electrical injury patients were matched with up to ten other patients with an eye injury (DT15 in the DNPR). We could not identify eye injuries in the DWEA register.

The matching variables were sex, year of injury and age. For all matches, the match-persons were randomly chosen, if more than ten were available per patient. This randomisation made it possible for the same person to act as a match-person for more than one electrical injury, but only in that particular year, as only the first event was used. If a person had both an electrical injury and a dislocation/sprain within the same year, the person could not be matched to him/herself. If it was impossible to match the exact age, the algorithm identified the closest person in age within the same 5 years’ age group, but of the same sex and injury-year.

The diagnoses of dislocation/sprain and eye injury were chosen since they are frequent, and are not suspected of causing the types of outcomes being studied.

*Match 3 –Occupation*. Electrical injury patients were matched with up to ten other persons from the working population, of the same occupation-group, sex and age. The patient and the match-persons were working at the time of the match. However, the electrical injury registered in the DNPR could have happened outside work. Match-persons were assigned a fictive injury date, based on their match-person’s injury, to be able to identify outcomes before and after a specific point in time. The purpose of this match was to determine whether persons with certain occupations presented a higher risk of the outcomes we were studying, due to socioeconomic factors or other occupational exposures than electrical shocks.

If a person had experienced more than one electrical injury during the period studied, only the first injury was included in this study. A person that had experienced an electrical injury was eligible as a match-person before and after the observation period (two years before and after the injury). Thus, one individual could be part of the data set more than once, if the time periods were separate. Persons with an injury that could not be matched with at least one match-person were excluded.

In the occupation match, all persons with an injury registered with the DWEA were defined as part of the working population, as their injury had occurred while working. However, not all were defined as part of the workforce by Statistics Denmark, probably because some had experienced their electrical injury while working a part-time job (students, interns or retired persons). Thus, 175 persons with an injury registered with the DWEA could not be matched in the occupation match, but only in the two injury matches with dislocation/sprain as controls.

#### Outcomes

We preselected a wide range of possible sequelae as outcomes, based on the literature, including reviews, original studies and case reports, and experience from clinical praxis at our department of occupational medicine. These outcomes were examined one by one. The outcomes related to the CNS with an ICD-10 diagnosis were: Spinal muscular atrophy and related syndromes (G12, G13), Parkinson’s disease (G20, G21, G22), Dystonia (G24), Essential tremor (G25.0), Other degenerative diseases of nervous system (G31, G32), Multiple sclerosis (G35), Epilepsy (G40, G41), Migraine (G43), Headache (G44, R51), Vertigo (H81, H82, R41), Convulsions, not elsewhere classified (R56) and Abnormal involuntary movements (R25).

Outcomes related to the PNS were: Facial nerve disorders (G50, G51), Nerve root and plexus disorders (G54, G55), Mononeuropathy, arm (G56), Mononeuropathy, leg (G57), Other mononeuropathy (G58, G59), Polyneuropathy (G62, G63, G64) and Disturbances of skin sensation (R20).

#### Statistical methods

We compared the matched groups using conditional logistic regression for a range of time periods (six months to five years). We also did a conditional Cox regression for analyses of the complete follow-up period, including a subgroup analysis for matches 1 and 2, with persons who were part of the workforce at the time of the injury. We tested the proportional hazard assumption with Schoenfeld’s residuals test. To simplify data analyses and maximise statistical power we analysed each diagnosis individually and did not look at differences in patterns in diagnoses for individuals or follow the course of diagnoses for individual patients or groups of patients. If persons (both electrical-injury patients and match-persons) were registered with the outcome of interest before the matching day, they were excluded. This was done separately for each outcome, to keep the persons in the data set to examine the other outcomes. If an electrical injury patient was excluded, all their matching controls were also excluded, whereas match-persons were excluded individually, keeping the remaining match-persons and the exposed person in the data set. This means that for each analysis of a specific outcome, the study sample was different. These numbers are presented in Table 1.

**Table 1:** Exclusions and diagnoses of electrical injured individuals for each of the three matches.

	Match 1: Dislocation/sprain			Match 2: Eye			Match 3: Occupation		
	(14.112 injuries)			(13.387 injuries)			(11.466 injuries)		
	Excluded due to previous outcome	Diagnosed during 5 years	Diagnosed during full follow-up	Excluded due to previous outcome	Diagnosed during 5 years	Diagnosed during full follow-up	Excluded due to previous outcome	Diagnosed during 5 years	Diagnosed during full follow-up
**Outcome**	n (%)	n (%)	n (%)	n (%)	n (%)	n (%)	n (%)	n (%)	n (%)
**Central nerve system:**									
Spinal muscular atrophy and related syndromes	<5	<5	6	<5	<5	6	<5	<5	<5
ICD-10: G12, G13	(NA)	(NA)	(NA)	(NA)	(NA)	(NA)	(NA)	(NA)	(NA)
Parkinson´s Disease	<5	<5	5	<5	<5	<5	0	<5	<5
ICD-10: G20, G21, G22	(NA)	(NA)	(NA)	(NA)	(NA)	(NA)	(0.0)	(NA)	(NA)
Essential tremor	<5	0	<5	<5	0	<5	<5	0	<5
ICD-10: G25.0	(NA)	(0.0)	(NA)	(NA)	(0.0)	(NA)	(NA)	(0.0)	(NA)
Other degenerative diseases of the nervous system	<5	<5	8	<5	<5	7	0	<5	5
ICD-10: G31, G32	(NA)	(NA)	(NA)	(NA)	(NA)	(NA)	(0.0)	(NA)	(0.0)
Multiple sclerosis	17	21	31	16	21	31	14	14	24
ICD-10: G35	(0.1)	(0.1)	(0.2)	(0.1)	(0.2)	(0.2)	(0.1)	(0.1)	(0.2)
Epilepsy	191	99	140	187	98	139	133	67	102
ICD-10: G40, G41	(1.4)	(0.7)	(1.0)	(1.4)	(0.7)	(1.1)	(1.2)	(0.6)	(0.9)
Migraine	183	97	181	172	90	172	134	72	142
ICD-10: G43	(1.3)	(0.7)	(1.3)	(1.3)	(0.7)	(1.3)	(1.2)	(0.6)	(1.3)
Headache	363	230	436	353	212	413	256	170	333
ICD-10: G44, R51	(2.6)	(1.7)	(3.2)	(2.6)	(1.6)	(3.2)	(2.2)	(1.5)	(3.0)
Vertigo	192	161	331	184	156	321	132	100	227
ICD-10: H81, H82, R42	(1.4)	(1.2)	(2.4)	(1.4)	(1.2)	(2.4)	(1.2)	(0.9)	(2.0)
Dystonia	6	8	16	6	8	16	5	7	14
ICD-10: G24	(0.0)	(0.1)	(0.1)	(0.0)	(0.1)	(0.1)	(0)	(0.1)	(0.1)
Convulsions, not elsewhere classified	132	49	84	126	47	81	92	32	56
ICD-10: R56	(0.9)	(0.4)	(0.6)	(0.9)	(0.4)	(0.6)	(0.8)	(0.3)	(0.5)
Abnormal involuntary movements	37	46	88	37	44	86	28	30	68
ICD-10: R25	(0.3)	(0.3)	(0.6)	(0.3)	(0.3)	(0.6)	(0.2)	(0.3)	(0.6)
**Peripheral nerve system:**									
Mononeuropathy, arm	214	202	357	199	176	330	169	158	281
ICD-10: G56	(1.5)	(1.5)	(2.6)	(1.5)	(1.3)	(2.5)	(1.5)	(1.4)	(2.5)
Mononeuropathy, leg	35	24	43	34	22	40	27	21	34
ICD-10: G57	(0.2)	(0.2)	(0.3)	(0.3)	(0.2)	(0.3)	(0.2)	(0.2)	(0.3)
Other mononeuropathy	5	<5	11	5	<5	11	<5	<5	8
ICD-10: G58, G59	(0)	(NA)	(0.1)	(0)	(NA)	(0.1)	(NA)	(NA)	(NA)
Polyneuropathy	18	25	55	17	23	52	13	13	37
ICD-10: G62, G63, G64	(0.1)	(0.2)	(0.4)	(0.1)	(0.2)	(0.4)	(0.1)	(0.1)	(0.3)
Facial nerve disorders	52	21	63	50	18	58	34	18	52
ICD-10: G50, G51	(0.4)	(0.1)	(0.4)	(0.4)	(0.1)	(0.4)	(0.3)	(0.2)	(0.5)
Nerve root and plexus disorders	25	18	26	23	16	24	19	15	23
ICD-10: G54, G55	(0.2)	(0.1)	(0.2)	(0.2)	(0.1)	(0.2)	(0.2)	(0.1)	(0.2)
Disturbances of skin sensation	23	28	60	21	27	59	16	17	38
ICD-10: R20	(0.2)	(0.2)	(0.4)	(0.2)	(0.2)	(0.4)	(0.1)	(0.1)	(0.3)

NA: not able to calculate

Due to Statistic Denmarks rules of reporting data, cells with less than five persons are given the same value: <5.

Electrical injury patients and match-persons who emigrated or died during follow-up were excluded from the date of this occurrence.

In sensitivity analyses, we used length of hospitalisation as a proxy for severity, given the probability that the most severe accidents would result in longer hospital stays. Therefore, we excluded patients who were hospitalised for less than one day, to determine whether the more severe injuries would reveal stronger associations with outcomes. As several authors have described traumatic brain injuries related to electrical injury [24, 25], we carried out additional analyses in which we excluded patients who were registered as diagnosed with concussion (S06.0) during the same hospitalisation as the electrical injury, to rule out a traumatic head injury being the cause of the outcome, that is, when a patient had fallen. In such cases, the outcomes could be related to post-commotional syndrome. For the outcomes related to the PNS, we adjusted for a previous diabetes diagnosis (E10-E14), as this could be a possible independent risk factor for the outcomes. Furthermore, we analysed whether persons who were diagnosed with *Epilepsy* after an electrical injury had been diagnosed with *Convulsions* (R56) before the injury, given the probability that convulsions could have caused the electrical injury, and indicate that the person already suffered from (undiagnosed) epilepsy at the time of the injury. Finally, to investigate possible late onset effects of the electrical injury, we carried out a supplementary analysis for the most frequent outcomes, where we excluded individuals who reported the outcome before each follow-up period. All sensitivity analyses were carried out in match 1 only.

## Results

We identified 20,155 electrical injuries in the DNPR and 1,810 in the DWEA register. After excluding persons under 18 years, persons without a valid CPR number and persons who died within the first 2 days of their accident, there was an overlap of 817 persons from the two registers. Invalid CPR numbers may reflect entries for tourists or migrant workers in the DNPR and DWEA registers, or possible mistyping in the DWEA register. When the overlap was eliminated and only the first electrical injury for each person kept, we had 14,112 injures (13,317 injuries from the DNPR and 795 from the DWEA prioritising DWEA registrations over DNPR if there was a double registration). These were used for match 1 (distorsion). For match 2 (eye) we identified 13,387 DNPR injuries. For match 3 (occupation) we excluded 2,646 persons who were not in the workforce. Here, we had 10,764 injuries from the DNPR and 702 from the DWEA register available for the occupation match. A match with 10 match-persons was possible for almost all electrical injuries, before the exclusions. A full flowchart of the study has been published previously in [26].

Most of the injuries happened to men (85.4% in DWEA and 76.4% in DNPR), and younger persons (<40 years) were overrepresented (60.3% in DWEA and 74.7% in DNPR). The occupations with most injuries were craft workers (50.1% in DWEA and 36.9% in DNPR), but service and sales workers were also overrepresented (8.8% in DWEA and 11.3% in DNPR), even when comparing to the distribution of occupations in Denmark. Only 1/5 of the injuries led to hospitalisation for one day or more (19.4% in DWEA and 21.9% in DNPR). More details on the study population can be found in [26].

The frequency of the examined outcomes varied greatly in our data set. Some of the diagnoses, such as *migraine*, *headache*, *vertigo* and *mononeuropathy in the arm* were common, whereas others were rare (Table 1). In particular, the outcomes of *spinal muscular atrophy and related syndromes*, *Parkinson’s disease*, *essential tremor*, *multiple sclerosis*, *dystonia*, *other degenerative diseases of the nervous system* and *other mononeuropathies* were rare, with fewer than 20 cases during full follow-up, and nearly none during the first five years.

The rarity of some of the outcomes affects the precision of the estimates of association, especially the estimates with short follow-ups, which are not always possible to estimate or have wide confidence intervals. We provide the results for these underpowered analyses, but encourage cautious interpretation (Table 2).

**Table 2:** Associations between electrical injuries and outcomes for the whole study period and in time intervals (electrical injuries matched in three different ways).

		Time to event	Time to event Workforce only	0–6 months	0–12 months	0–2 years	0–3 years	0–4 years	0–5 years
Outcome	Match	HR	HR	OR	OR	OR	OR	OR	OR
**Central nerve system:**									
Spinal muscular atrophy and related syndromes	1 Dislocation/sprain	1.14	1.36	*	*	6.67	2.50	1.81	1.25
		[0.41;3.22]	[0.30;6.24]			[1.11;39.90]	[0.53;11.77]	[0.40;8.20]	[0.29;5.43]
ICD-10: G12, G13	2 Eye	1.21	1.03	20.00	6.67	2.86	2.00	1.54	1.43
		[0.43;3.42]	[0.23;4.51]	[1.81;220.56]	[1.11;39.90]	[0.59;13.75]	[0.44;9.13]	[0.35;6.82]	[0.32;6.29]
	3 Occupation		1.00	*	*	*	*	*	*
			[0.23;4.28]						
Parkinson´s Disease	1 Dislocation/sprain	0.78	0.94	*	*	*	4.00	3.33	2.00
ICD-10: G20, G21, G22		[0.28;2.16]	[0.27;3.29]				[0.78;20.62]	[0.90;12.31]	[0.58;6.91]
	2 Eye	0.64	0.42	*	*	*	1.25	2.22	1.25
		[0.20;2.05]	[0.10;1.81]				[0.16;9.99]	[0.48;10.28]	[0.28;5.44]
	3 Occupation		1.03	*	*	*	2.50	1.42	1.43
			[0.32;3.40]				[0.53;11.77]	[0.32;6.29]	[0.32;6.29]
Essential tremor	1 Dislocation/sprain	1.03	0.91	*	*	*	*	*	*
ICD-10: G25.0		[0.32;3.40]	[0.21;3.96]						
	2 Eye	0.93	0.92	*	*	*	*	*	*
		[0.29;3.05]	[0.21;4.08]						
	3 Occupation		1.00	*	*	*	*	*	*
			[0.23;4.26]^						
Other degenerative diseases of the nervous system	1 Dislocation/sprain	0.81	1.01	*	*	*	*	0.30	0.77
		[0.37;1.75]	[0.35;2.86]					[0.04;2.22]	[0.24,2.49]
ICD-10: G31, G32	2 Eye	0.81	1.01	*	*	*	*	0.30	0.77
		[0.37;1.75]	[0.35;2.86]					[0.04;2.22]	[0.24,2.49]
	3 Occupation		1.66	*	*	*	*	*	2.50
			[0.65;4.30]						[0.53;11.77]
Multiple sclerosis	1 Dislocation/sprain	0.99	0.96	0.62	1.30	0.98	1.08	1.11	1.02
ICD-10: G35		[0.62;1.56]	[0.56;1.64]	[0.08;4.68]	[0.39;4.33]	[0.39;2.45]	[0.54;2.15]	[0.60;2.07]	[0.56;1.84]
	2 Eye	1.13	0.92	1.43	2.00	1.56	1.58	1.39	1.22
		[0.71;1.79]	[0.54;1.59]	[0.18;11.61]	[0.58;6.91]	[0.61;4.01]	[0.78;3.18]	[0.74;2.62]	[0.67;2.23]
	3 Occupation		1.02	1.43	0.59	0.75	0.94	0.91	0.91
			[0.60;1.73]	[0.18;11.61]	[0.08;4.42]	[0.23;2.42]	[0.38;2.36]	[0.39;2.10]	[0.42;1.97]
Epilepsy	1 Dislocation/sprain	1.13	1.35	1.27	1.14	1.54	1.42	1.42	1.37
ICD-10: G40, G41		[0.90;1.43]^	[1.02;1.77]^	[0.50;3.28]	[0.57;2.27]	[1.02;2.33]	[1.00;2.02]	[1.04;1.93]	[1.03;1.83]
	2 Eye	1.26	1.31	1.37	1.06	1.79	1.57	1.50	1.53
		[1.00;1.59]^	[1.00;1.73]^	[0.54;3.48]	[0.51;2.20)	[1.11;2.60]	[1.10;2.25]	[1.10;2.05]	[1.14;2.05]
	3 Occupation		1.55	1.58	1.71	2.30	1.99	2.05	1.97
			[1.18;2.03]^	[0.47;5.34]	[0.72;4.06]	[1.40;3.79]	[1.29;3.05]	[1.40;3.01]	[1.39;2.79]
Migraine	1 Dislocation/sprain	1.51	1.59	1.82	1.33	1.57	1.44	1.52	1.46
ICD-10: G43		[1.28;1.79]	[1.32;1.93]	[0.96;3.47]	[0.81;2.17]	[1.12;2.20]	[1.08;1.91]	[1.19;1.94]	[1.16;1.85]
	2 Eye	1.55	1.58	1.75	1.60	1.68	1.57	1.69	1.58
		[1.30;1.85]	[1.29;1.94]	[0.89;3.44]	[0.96;2.67]	[1.18;2.38]	[1.16;2.11]	[1.30;2.20]	[1.24;2.02]
	3 Occupation		2.14	2.49	2.23	2.30	2.28	2.39	2.10
			[1.76;2.60]	[1.08;5.74]	[1.22;4.07]	[1.52;3.47]	[1.63;3.19]	[1.79;3.21]	[1.60;2.76]
Headache	1 Dislocation/sprain	1.41	1.47	2.04	1.71	1.62	1.45	1.42	1.43
ICD-10: G44, R51		[1.27;1.57]	[1.29;1.66]	[1.38;3.02]	[1.26;2.32]	[1.32;2.00]	[1.21;1.74]	[1.29;1.66]	[1.23;1.65]
	2 Eye	1.32	1.29	1.50	1.35	1.31	1.19	1.22	1.27
		[1.18;1.47]	[1.14;1.47]	[0.97;2.34]	[0.97;1.87]	[1.05;1.63]	[0.98;1.44]	[1.04;1.45]	[1.09;1.48]
	3 Occupation		1.95	2.78	1.89	2.18	2.00	1.97	2.04
			[1.72; 2.21]	[1.71;4.51]	[1.30;2.74]	[1.69;2.80]	[1.61;2.48]	[1.63;2.39]	[1.71;2.42]
Vertigo	1 Dislocation/sprain	1.53	1.44	1.66	1.78	1.62	1.81	1.66	1.70
ICD-10: H81, H82, R42		[1.35;1.74]	[1.24;1.68]	[0.98;2.83]	[1.23;2.59]	[1.23;2.12]	[1.46;2.24]	[1.37;2.02]	[1.43;2.02]
	2 Eye	1.59	1.52	2.05	1.79	1.57	1.83	1.67	1.73
		[1.40;1.81]	[1.30;1.78]	[1.18;3.58]	[1.22;2.62]	[1.19;2.07]	[1.47;2.28]	[1.37;2. 04)	[145;2.07)
	3 Occupation		1.95	1.78	1.99	1.69	1.94	1.92	2.01
			[1.68;2.28]	[0.88;3.63]	[1.23;3.23]	[1.18;2.43]	[1.47;2.56]	[1.49;2.46]	[1.61;2.51]
Dystonia	1 Dislocation/sprain	1.59	1.97	*	0.71	0.90	0.55	1.11	1.29
ICD-10: G24		[0.92;2.74]	[1.06;3.66]		[0.09;5.39]	[0.21;3.85]	[0.13;2.29]	[0.44;2.79]	[0.59;2.84]
	2 Eye	1.44	1.58	*	0.50	0.74	0.59	1.22	1.30
		[0.84;2.48]	[0.87;2.88]		[0.07;3.73]	[0.18;3.11]	[0.14;2.45]	[0.48;3.09]	[0.59;2.85]
	3 Occupation		2.50	*	2.45	2.20	1.32	1.66	2.21
			[1.36;4.58]		[0.27;21.90]	[0.48;10.19]	[0.30;5.79]	[0.58;4.78]	[0.91;5.36]
Convulsions, not elsewhere classified	1 Dislocation/sprain	1.11	1.17	1.66	1.53	1.25	1.17	1.02	1.08
		[0.86;1.43]	[0.85;1.62]	[0.70;3.94]	[0.78;2,98]	[0.78;2.02]	[0.78;1.76]	[0.70;1.49]	[0.77;1.51]
ICD-10: R56									
	2 Eye	1.37	1.54	2.65	2.09	1.96	1.66	1.65	1.46
		[1.08;1.75]	[1.16;2.05]	[1.27;5.52]	[1.17;3.72]	[1.28;3.02]	[1.14;2.42]	[1.17;2.31]	[1.05;2.03]
	3 Occupation		1.45	3.51	3.55	2.32	2.01	1.73	1.66
			[1.06;2.00]	[1.38;8.91]	[1.66;7.61]	[1.29;4.14]	[1.19;3.39]	[1.08;2.78]	[1.08;2.56]
Abnormal involuntary movements	1 Dislocation/sprain	1.23	1.68	3.45	2.64	2.01	1.62	1.46	1.31
		[0.97;1.56]	[1.27;2.22]	[1.62,7.36]	[1.46;4.75]	[1.32;3.07]	[1.12;2.35]	[1.05;2.02]	[0.95;1.80]
ICD-10: R25									
	2 Eye	1.35	1.24	1.99	1.65	1.84	1.38	1.32	1.43
		[1.04;1.76]	[0.89;1.72]	[0.76;5.19]	[0.78;3.49]	[1.10;3.08]	[0.90;2.12]	[0.89;1.95]	[1.01;2.04]
	3 Occupation		2.17	6.36	3.81	3.54	2.91	3.10	2.47
			[1.64;2.87]	[2.47;16.42]	[1.69;8.60]	[2.03;6.15]	[1.79;4.74]	[2.01;4.77]	[1.62;3.77]
**Peripheral nerve system:**									
Mononeuropathy, arm	1 Dislocation/sprain	1.26	1.30	1.69	1.40	1.38	1.40	1.40	1.42
ICD-10: G56		[1.12;1.41]	[1.13;1.48]	[1.05;2.71]	[0.98;1,99]	[1.09;1.76]	[1.15;1.72]	[1.17;1.67]	[1.21;1.67]
	2 Eye	1.03	1.02	1.14	0.93	1.06	1.12	1.11	1.10
		[0.91;1.16]	[0.89;1.17]	[0.69;1.88]	[0.63;1.35]	[0.82;1.37]	[0.90;1.39)	[0.92;1.34]	[0.93;1.30]
	3 Occupation		1.41	1.99	1.53	1.65	1.61	1.59	1.57
			[1.24;1.61]	[1.16;3.40]	[1.04;2.25]	[1.26;2.16]	[1.29;2.03]	[1.30;1.94]	[1.31;1.89]
Mononeuropathy, leg	1 Dislocation/sprain	0.84	0.85	1.78	1.47	1.13	1.08	1.00	0.90
ICD-10: G57		[0.61;1.17]	[0.59;1.22]	[0.69;4.60]	[0.73;2.96]	[0.65;1.97]	[0.67;1.74]	[0.64;1.56]	[0.58;1.39]
	2 Eye	1.12	1.10	2.78	2.72	1.96	1.59	1.38	1.20
		[0.80;1.59]	[0.75;1.63]	[1.03;7.48]	[1.30;5.68]	[1.08;3.56]	[0.97;2.62]	[0.86;2.24]	[0.75;1.90]
	3 Occupation		1.46	2.64	3.18	2.83	2.31	1.83	1.66
			[1.01;2.12]	[0.87;7.94]	[1.43;7.05]	[1.49;5.38]	[1.34;3.97]	[1.11;3.02]	[1.02;2.80]
Other mononeuropathy	1 Dislocation/sprain	2.04	1.60	*	*	*	1.90	1.38	1.18
		[1.07;3.90]	[0.74;3.45]				[0.65;5.55]	[0.48;3.92]	[0.42;3.32]
ICD-10: G58, G59									
	2 Eye	1.80	1.55	*	*	*	1.82	1.60	1.29
		[0.95;3.43]	[0.72;3.31]				[0.63;5.28]	[0.56;4.60]	[0.46;3.66]
	3 Occupation		2.86	*	*	*	4.29	2.73	2.14
			[1.30;6.27]				[1.11;16.57]	[0.76;9.78]	[0.62;7.46]
Polyneuropathy	1 Dislocation/sprain	0.89	0.92	1.90	1.92	1.30	1.03	0.97	0.88
ICD-10: G62, G63, G64		[0.66;1.19]	[0.65;1.31]	[0.65;5.52]	[1.01;3.66]	[0.75;2.28]	[0.62;1.69]	[0.61;1.54]	[0.57;1.36]
	2 Eye	1.10	1.10	2.67	3.13	2.16	1.83	1.44	1.24
		[0.81;1.50)	[0.75;1.60]	[0.89;8.03]	[1.54;6.36]	[1.19;3.93]	[1.08;3.12]	[0.88;2.36]	[0.78;1.98]
	3 Occupation		1.23	1.25	2.78	1.95	1.49	1.43	1.17
			[0.86;1.76]	[0.16;9.99]	[1.03;7.48]	[0.91;4.16]	[0.77;2.90]	[0.78;2.62]	[0.64;2.12]
Facial nerve disorders	1 Dislocation/sprain	1.11	1.12	2.49	1.55	1.21	0.85	0.76	0.74
ICD-10: G50, G51		[0.85;1.45]^	[0.83;1.52]^	[0.93;6.63]	[0.70;3.43]	[0.69;2.11]	[0.51;1.42]	[0.47;1.22]	[0.48;1.16]
	2 Eye	1.10	1.21	1.72	1.13	1.09	0.87	0.74	0.67
		[0.83;1.45]^	[0.87;1.67]^	[0.59;4.97]	[0.48;2.62]	[0.60;1.97]	[0.51;1.51]	[0.45;1.23]	[0.42;1.08]
	3 Occupation		1.39	2.73	1.66	1.71	1.37	1.11	1.02
			[1.03;1.88]^	[0.76;9.78]	[0.65;4.30]	[0.93;3.16]	[0.78;2.40]	[0.66;1.86]	[0.63;1.67]
Nerve root and plexus disorders	1 Dislocation/sprain	0.92	1.00	3.32	3.20	1.81	1.73	1.34	1.46
ICD-10: G54, G55		[0.61;1.39]^	[0.64;1.57]^	[1.20;9.12]	[1.51;6.77]	[0.92;3.55]	[0.96;3.11]	[0.77;2.34]	[0.89;2.39]
	2 Eye	0.86	0.93	3.55	3.47	1.21	1.22	1.09	1.32
		[0.56;1.32]^	[0.59;1.49]	[1.28;9.86]	[1.55;7.75]	[0.58;2.52]	[0.65;2.28]	[0.59;1.96]	[0.78;2.22]
	3 Occupation		1.25	5.00	3.16	2.26	1.86	1.67	1.81
			[0.80;1.94]^	[1.51;16.60]	[1.26;7.91]	[0.99;5.13]	[0.94;3.65]	[0.88;3.16]	[1.04;3.14]
Disturbances of skin sensation	1 Dislocation/sprain	1.38	1.18	6.00	3.70	2.35	1.66	1.52	1.55
ICD-10: R20		[1.03;1.84]	[0.82;1.71]	[2.18;16.51]	[1.79;7.65]	[1.36;4.05]	[1.01;2.74]	[0.97;2.37]	[1.04;2.30]
	2 Eye	1.33	1.07	3.75	2.61	2.37	1.92	1.74	1.71
		[0.99;1.77]	[0.74;1.54]	[1.47;9.58]	[1.30;5.23]	[1.35;4.16]	[1.14;3.23]	[1.09;2.77]	[1.14;2.58]
	3 Occupation		1.34	4.55	4.38	2.13	1.51	1.43	1.37
			[0.93;1.92]	[1.58;13.08]	[1.80;10.63]	[1.08;4.21]	[0.80;2.84]	[0.80;2.55]	[0.82;2.28]

HR: Hazard Ratio.

OR: Odds Ratio.

* Too few events to estimate risk.

^Proportional hazard not present.

For the diagnoses related to the CNS, we found clear patterns of association with previous electrical injury for *epilepsy*, *migraine*, *headache* and *vertigo* across almost all match groups and time periods, with odds ratios of about 1.5 to 2.0, and similar hazard ratios in the time-to-event analyses (Table 2). The patterns of association were a little less clear for *convulsions* and *abnormal involuntary movements*, but generally showed odds ratios between 2.0 and 4.0, and hazard ratios of about 1.5 to 2.0 in the time-to-event analyses. We found an association with *spinal muscular atrophy* with high odds ratios in match 2 at 6 and 12 months, however, this was based on very few cases. This was also the case for *dystonia*, where the time-to-event analysis showed a hazard ratio of about 2 in matches 1 and 3, whereas no associations were apparent in the analyses of specific time periods. Thus, for both these diagnoses the associations are uncertain, due to the small number of cases available for the analyses. We did not find any associations between electrical injuries and *Parkinson’s disease*, *essential tremor*, *multiple sclerosis* or *other degenerative diseases of the nervous system*, which were also rare outcomes in our data (see Table 1).

For the diagnoses related to the PNS, we found odds ratios of about 4 to 6 across match groups for *disturbances of skin sensation* in the first 6 months after an electrical injury, which decreased at later points in time. The pattern of associations was somewhat the same for *nerve root and plexus disorders*, where the odds ratios were about 3 to 5 across match groups at 6 and 12 months, and decreased in the analyses of longer time periods, whereas no increased risk was seen in the time-to-event analysis. We also found an increased risk of *mononeuropathy* in the arm or leg. For *mononeuropathy in the arm*, the increased risk was identified in matches 1 and 3, but not in match 2. There were odds ratios of 1.7 to 2.0 at 6 months, which decreased to about 1.5 at later dates, and there was a hazard ratio of about 1.3 to 1.4 in the time-to-event analyses. *Mononeuropathy in the leg* was rarer, and here we identified the increased risk in matches 2 and 3, but not in match 1. There were odds ratios of 2.6 to 3.0 at 6 and 12 months, which decreased in the analyses at later dates, and no increased risk was seen in the time-to-event analysis. For the remaining three diagnoses related to the PNS, the patterns of associations with electrical injury were less certain, probably due to the rarity of the cases. *Other mononeuropathy* showed an increased risk in the time-to-event analysis for match 3, with a hazard ratio of 2.9 and an odds ratio of 4.3, with a wide confidence interval at 3 years. For *facial nerve disorders*, the time-to-event analysis showed an increased risk for match 3, whereas no associations were seen in analyses at specific times. The time-to-event analysis showed no increased risk of *polyneuropathy*, but for matches 2 and 3 there was an increased risk at 2 and 3 years, with an odds ratio of about 2.0 to 3.0.

### Sensitivity analyses

Restricting the time-to-event analyses to patients in the workforce did not significantly change most of the estimates (Table 2). When we restricted analysis to injuries that resulted in hospitalisation lasting longer than one day, most estimates increased (Table 3). The estimates did not change when we adjusted for diabetes in the analyses of the outcomes related to the PNS, or when we excluded the few with a *concussion* diagnosis from the analyses of the outcomes related to the CNS (Table 3). For patients who were diagnosed with *epilepsy*, the occurrence of *convulsions* preceding the injury was no different in the group that suffered an electrical injury (18.4%[12.1–24,6%]) than among matched persons 17.1%[15.2–19.0%]).

**Table 3:** Sensitivity analyses (electrical injuries matched with dislocation/sprain controls). Stratified on length of hospitalisation, adjusted for diabetes and with exclusion for concussion in connection to the injury.

	Time to event from Table 2, (Dislocation/sprain match)	Length of hospitalisation < = 1 day	Length of hospitalisation >1 day	Adjusted for diabetes	Concussion excluded
Outcome	HR	HR	HR	HR	HR
**Central nerve system:**					
Spinal muscular atrophy and related syndromes	1.14	1.29	2.50		1.58
	[0.41;3.22]	[0.51;3.28]	[0.71;8.86]		[0.67;3.73]
Parkinson´s Disease	0.78	0.62	1.18		0.72
	[0.28;2.16]	[0.22;1.69]	[0.27;5.09]		[0.29;1.80]
Essential tremor	1.03	0.32	3.33		0.97
	[0.32;3.40]	[0.04;2.34]	[0.67;16.51]		[0.30;3.17]
Other degenerative diseases of the nervous system	0.81	0.90	0.28		0.87
	[0.37;1.75]	[0.44;1.86]	[0.04;2.01]		[0.42;1.80]
Multiple sclerosis	0.99	0.82	0.89		0.83
	[0.62;1.56]	[0.56;1.20]	[0.45;1.76]		[0.57;1.20]
Epilepsy	1.13	0.89	0.93		0.92
	[0.90;1.43]	[0.74;1.07]	[0.66;1.31]		[0.76;1.10]
Migraine	1.51	1.50	1.93		1.50
	[1.28;1.79]	[1.27;1.76]	[1.42;2.62]		[1.28;1.76]
Headache	1.41	1.38	1.66		1.38
	[1.27;1.57]	[1.24;1,.53]	[1.37;2.01]		[1.25;1.53]
Vertigo	1.53	1.54	1.57;1.25;1.97]		1.54
	[1.35;1.74]	[1.36;1.74]			[1.37;1.74]
Dystonia	1.59	1.20	2.92		1.35
	[0.92;2.74]	[0.69;2.09]	[1.26;6.77]		[0.80;2.28]
Convulsions, not elsewhere classified	1.23	1.10	1.34		1.18
	[0.97;1.56]	[0.87;1.40]	[0.89;2.02]		[0.94;1.47]
Abnormal involuntary movements	1.11	1.18	1.26		1.14
	[0.86;1.43]	[0.93;1.48]	[0.83;1.93]		[0.90;1.44]
**Peripheral nerve system:**					
Mononeuropathy, arm	1.26	1.25	1.38	1.27	
	[1.12;1.41]	[1.12;1.40]	[1.12;1.69]	[1.13;1.42]	
Mononeuropathy, leg	0.84	0.86	0.89	0.86	
	[0.61;1.17]	[0.62;1.18]	[0.49;1.60]	[0.63;1.19]	
Other mononeuropathy	2.04	2.03	2.94	2.00	
	[1.07;3.90]	[1.06;3.88]	[1.09;7.97]	[1.05;3.82]	
Polyneuropathy	0.89	0.86	1.40	0.93	
	[0.66;1.19]	[0.64;1.16]	[0.91;2.17]	[0.70;1.23]	
Facial nerve disorders	1.11	1.05	1.27	1.06	
	[0.85;1.45]	[0.80;1.38]	[0.79;2.05]	[0.81;1.38]	
Nerve root and plexus disorders	0.92	0.86	1.18	0.92	
	[0.61;1.39]	[0.56;1.32]	[0.59;2.36]	[0.61;1.39]	
Disturbances of skin sensation	1.38	1.38	2.08	1.38	
	[1.03;1.84]	[1.03;1.84]	[1.30;3.32]	[1.04;1.84]	

We were able to estimate possible late onset effects for the seven most frequent outcomes (Table 4). For the diagnoses related to the CNS, we found no increased risk of Epilepsy within the first 12 months after the injury. Thereafter the risk increased with the highest risk during the 12–24 months’ time interval (odds ratio 1.62), whereafter the risk stabilised at about 1.4 in the following time intervals. Migraine and headache showed similar patterns, where the highest odds ratios (about 1.8–2.0) were in the first 6 months after the injury, whereafter the risk stabilised at a lower level (about 1.3–1.4) in the following time intervals. Vertigo showed an increased risk in all time intervals with odds ratios from 1.6 to 1.9. An increased risk within the first 6 months after the injury was also found for *Abnormal involuntary movements* with an odds ratio of 3.45, while there was no increased risk in the later time intervals. There was no increased risk for *Convulsions* in any time intervals, although the odds ratio was highest in the first 6 months.

**Table 4:** Associations between electrical injuries and outcomes for separate time intervals.

	0-<6 months	06-<12 months	12-<24 months	2-<3 years	3-<4 years	4–5 years
Outcome*	OR	OR	OR	OR	OR	OR
**Central nerve system**						
Epilepsy	n = 22	n = 9	n = 29	n = 19	n = 12	n = 8
	1.27[0.50;3.23]	1.01[0.36;2.83]	1.62[1.02;2.57]	1.45[0.99;2.11]	1.44[1.04;2.00]	1.38[1.02;1.87]
Migraine	n = 15	n = 7	n = 25	n = 21	n = 18	n = 11
	1.82[0.96;3.47]	0.93[0.43;2.02]	1.49[1.00;2.23]	1.37[1.00;1.87]	1.48[1.13;1.93]	1.42[1.11;1.82]
Headache	n = 33	n = 24	n = 59	n = 37	n = 40	n = 37
	2.04[1.38;3.02]	1.36[0.84;2.19]	1.49[1.16;1.91]	1.34[1.09;1.64]	1.33[1.11;1.58]	1.36[1.16;1.59]
Vertigo	n = 20	n = 18	n = 30	n = 41	n = 23	n = 29
	1.66[0.98;2.83]	1.93[1.15;3.25]	1.61[1.18;2.21]	1.84[1.46;2.33]	1.66[1.35;2.05]	1.70[1.42;2.05]
Convulsions, not elsewhere classified	n = 12	n = 5	n = 10	n = 8	n = 5	n = 9
	1.66[0.70;3.94]	1.37[0.48;3.88]	1.12[0.63;2.00]	1.08[0.68;1.71]	0.93[0.61;1.42]	1.01[0.70;1.46]
Abnormal involuntary movements	n = 12	n = 5	n = 13	n = 9	n = 8	n = 5
	3.45[1.62;7.36]	1.85[0.71;4.81]	1.65[0.99;2.75]	1.35[0.88;2.08]	1.25[0.87;1.81]	1.24[0.79;1.69]
**Peripheral nerve system**						
Mononeuropathy, arm	n = 32	n = 20	n = 44	n = 39	n = 34	n = 33
	1.69[1.05;2.71]	1.13[0.66;1.93]	1.29[0.98;1.72]	1.35[1.08;1.68]	1.36[1.12:1.64]	1.39[1.17;1.64]

* Presented only for outcomes with sufficient number of cases to display in time intervals, and for match 1 (dislocation/strain)

We were only able to estimate late onset effects for one diagnosis related to the PNS, and here we found and increased risk for Mononeuropathy in the arm within the first six months, whereafter the risk stabilised at a lower level (about 1.3–1.4) in the following time intervals.

## Discussion

To the best of our knowledge, this study is the first to examine the association between electrical injuries and conditions related to the CNS and PNS, through a register-based, prospective cohort study that uses a matched design. We studied 14,112 electrical injuries identified over a period of 19 years, and found an increased risk of conditions related to both the CNS and PNS in the years following an electrical injury, when compared with three different matched control groups.

For the CNS, we identified an increased risk of *epilepsy*, *convulsions*, *abnormal involuntary movements*, *headache*, *migraine* and *vertigo*. We also identified an uncertain, increased risk of *spinal muscular atrophy* and *dystonia*, whereas no increased risk was identified for *Parkinson’s disease*, *essential tremor*, *multiple sclerosis* or *other degenerative diseases in the nervous system*.

For the PNS, we identified an increased risk of *disturbances of skin sensation*, *mononeuropathy in the arm* or *leg* and *nerve root and plexus disorders*. We also identified an uncertain, increased risk of *facial nerve disorders*, *other mononeuropathy* and *polyneuropathy*.

Our sensitivity analyses revealed that the risk estimates increased when we restricted our analyses to injuries that required hospitalisation of longer than one day as a proxy for severity, whereas other sensitivity analyses did not affect the estimates.

### Limitations

Because this is a register study, there are some inherent risks of bias that should be addressed. Registration in the DNPR is probably incomplete and thus only a proportion of the electrical injuries were registered. This may indicate underreporting, especially during the first years of the period studied. If the types of electrical injuries registered differ in type, severity or duration from those not registered, this may cause bias in an unknown direction. Even today, underreporting may be a problem, since detailed registration in the casualty departments may be deprioritised in acute situations. If the electrical injuries not reported in the DNPR were the most severe, where the consequence of the injury, such as a burn, was registered, and not the code for the electrical injury itself, we may have overlooked the most severe injuries, and thus underestimated the associations between the injuries and our outcomes. In the part of the cohort derived from the DWEA register, we have no reason to think that there is any difference in reporting due to exposure, although the number of injuries decreased over time, which is also the case in other types of work injuries [27].

The severity and other characteristics of the electrical injuries were not registered, since the definition of an electrical injury was based on the ICD-10 code in the DNPR, and type of injury, in the DWEA register. Previous studies have distinguished between high- and low-voltage injuries as a measure of severity. We tried to accommodate this by restricting our analysis to patients who were hospitalised for longer than one day as a proxy for severity, and found that most estimates of association with outcomes increased. This indicates that the severity of an injury is positively related to the risk of developing a CNS or PNS disorder in the years following the injury. In most cases, hospitalisation was very brief, and a large proportion of electrical injuries registered with the DWEA did not involve hospitalisation, or involved only an outpatient visit. The definition of an occupational accident that should be registered with the DWEA is an accident that has led to at least one day of sick leave in addition to the day of the accident, and thus of a certain severity.

The outcomes used in the study are diagnoses from the DNPR register which covers all hospital contacts. However, some of the symptoms and diagnoses used in the study would probably not require a hospital contact but could instead be handled in the primary care sector. So, it is possible that some patients were seen by their general practitioners during follow-up, and thus not registered in the DNPR. A previous study in the same population confirms this, as persons with an electrical injury had an increased risk of many contacts with general practitioners in the five years following the injury compared to matched controls [28]. Consequently, our analyses based on DNPR data underestimates the risk of getting the symptoms and diagnoses that would normally primarily be handled in the primary care sector, such as headache or migraine.

A further potential issue with the outcomes is that some of the persons may have been diagnosed with the outcomes of interest prior to 1994, and thus should have been excluded from the study, However, this is only an issue if persons diagnosed prior to 1994, were not seen at a hospital due to their disease/disorder in the time period after 1994 and before their injury (a period ranging from 2–20 years). Several of the diagnoses under study, such as epilepsy, require regular hospital contacts and we would therefore be able to identify and exclude them. If there is an issue, it would be non-differential between persons with electrical injuries and match persons. Thus, if present, the bias would cause us to overlook associations.

An important further limitation was the choice of matched persons. We found it difficult to identify the perfect type of injury to match with an electrical injury. Since the electrical injuries are heterogeneous in severity, ideally, the match group would be similarly heterogeneous, and at the same time, it would have a rather frequently-occurring type of injury, to enable us to find a sufficient number of suitable matched persons. Our solution was to use three different types of matching: patients with a dislocation/sprain, patients with eye injuries and persons from the same occupation group as those with electrical injuries. The first two matches had the disadvantage that the injuries themselves were not life-threatening or disabling, as an electrical injury may be. Match 3, persons with the same type of job, had the disadvantage that the matched persons had no injury, and thus were probably not using the healthcare system at the time of the match. This means that the estimates based on match 3 (which were generally higher than the other two matches) are probably overestimated, if patients with electrical injuries had other healthcare seeking habits. In this case, we could not accommodate the possible bias by restricting the analyses based on length of hospitalisation, as this was not applicable to the matched control group. This same approach was used in a Danish cohort study on cardiac disease and mortality after electrical injuries which matched injured persons with randomly selected controls from the general population based on age and gender [29]. We aimed to match with other injured persons (matches 1 and 2) to avoid using overly healthy controls, but also to take into account socioeconomic position, when matching with occupation controls (match 3).

Misclassification also presents a risk of bias in this study. A range of the chosen diagnoses were symptoms, such as essential tremor, headache and vertigo. The diagnoses were based on the patients’ subjective complaints, and although they were real ailments and problems for the patients, they may not indicate specific diseases, and thus may be misclassified. Although the risk of misclassification may be highest when it comes to common symptoms, the impact on our results and conclusions would potentially be larger when it comes to rare diagnoses, where (lack of) association may be due to there being only a few cases, and thus being more vulnerable to random misclassification of one or two cases. Related to this issue, we retrieved outcome data from DNPR and the validity of these data are critical for our results. Previous studies have found a positive predictive value (PPV) of 81% of diagnoses related to the medical speciality in general [30] and specifically 69.9% for ALS, 82.4% for Parkinson’s disease, 95.1% for Multiple sclerosis, and 81.4% for Epilepsy [31]. A low PPV would indicate that too few people were diagnosed with the outcome, and thus limit our ability to identify an association, especially for the rare outcomes. However, it should not have an impact on the size of the risk estimates, as a low PPV would apply equally for persons with electrical injuries and matched individuals.

Finally, the size of this study was the largest possible using Danish data, but it still raises concerns about statistical power, with respect to some outcomes. Even though the DNPR was established in 1977, information about electrical injuries was insufficiently registered until the introduction of ICD-10 codes in 1994. Since we chose to include two years of observation time preceding the reported accidents to exclude persons with the outcome of interest, and at least two years of observation time following the injury, we were limited to 19 years, from 1996 to 2014. Despite the length of this period and the considerable number of injuries registered, we still had limited power in some analyses, especially with rare diagnoses and when the sequelae of interest had occurred prior to the injury. The latter could cause bias if a previously unregistered electrical injury was related to the outcome. However, since the matching included the year in which the injury occurred, the risk of missing previous injuries was the same for persons with electrical injuries and matched persons, and thus the risk of bias is unlikely. The statistical power, as well as lack of available information, also limited our possibilities to adjust for additional potential confounders, such as life style or personality factors.

### Diagnoses related to the CNS

Our results regarding conditions related to the CNS both confirm and conflict with previous research. First, our results confirm the increased risk of *vertigo* and *migraine*, which was previously identified in a Danish register study [4] that is partly based on some of the same data as this study, and also adds an increased risk of *headache*, which has also been seen in previous research [1, 8], and may be somewhat connected to migraine. None of these conditions have been studied extensively in relation to electrical injuries, although vertigo has been described in a few case reports [1].

Secondly, we found a greater risk of *epilepsy* following an electrical injury, which is consistent with previous studies [2, 4]. If electrical injuries cause epilepsy, one would expect an increased prevalence of epilepsy among electrical workers. However, a nationwide Swedish register study of the association between epilepsy and occupation did not find an increased prevalence among electrical workers [32]. Thus, reverse causation might be at play, as people with epilepsy are more prone to accidents of all kinds than people without epilepsy [33]. Hence, the injured persons may have had undiagnosed epilepsy before their electrical injury, and the injury may have been due to an epileptic seizure. To investigate this, we did a sensitivity analysis to see whether the injured persons had been diagnosed with *convulsions* before the electrical injury, as this may be an indication of *epilepsy*. However, our results showed no difference in the incidence of *convulsions* before the electrical injury in the group that suffered electrical injuries and matched persons. At the same time, we also found an increased risk of *convulsions* following an electrical injury. The same goes for the somewhat related diagnoses, *abnormal involuntary movements*, which also includes convulsive movements. So, it seems reasonable to assume that electrical injuries indeed may increase the risk of *epilepsy*, although a complex relationship may be at work, and further studies are needed to determine the mechanisms underlying the association.

Thirdly, our study did not specifically address the previously suggested association between electrical injury and ALS [2, 34], as ALS is very rare. Instead we focused on the higher level diagnoses of *spinal muscular atrophy and related syndromes*, which includes ALS, where we identify an uncertain association, due to a very small number of cases. However, previous reports have highlighted the delay of onset [2], which is the opposite of what our results show, where the possible association is most prominent in the shortest time periods, whereas the time-to-event analyses show no association. This suggests that the possible association is based on a few cases diagnosed shortly after an electrical injury. Thus, it seems most plausible that the results we found are coincidental, and not signs of a causal link. Recent reviews and meta-analyses have come to conflicting conclusions, where some conclude that ALS is associated with occupational exposure to magnetic fields, but not to electric shock [35, 36] whereas another highlights a history of electric shock as a risk factor for ALS [37]. Our data set did not include enough cases to shed further light on this question.

The remaining diagnoses related to the CNS also had the issue of few available cases in our population, which limited our ability to identify associations. Thus, we did not find an increased risk of *Parkinson’s disease*, *essential tremor* or *other degenerative diseases of the nervous system*. With regard to Parkinson’s disease, our results were consistent with a previous Danish register study [4] and a case-control study [3]. However, and also consistent with previous research [2], we did identify an uncertain association for the related movement disorder, *Dystonia*, in the time-to-event analysis, indicating that it may develop over time, but again, this diagnosis was rare in our population. *Multiple Sclerosis* was the last diagnosis for which we found no association with electrical injuries, which confirms the results of the previous Danish register study [4].

### Diagnoses related to the PNS

When we looked at the results related to conditions of the PNS, we found a rather clear pattern that showed that peripheral nerve disorders are common sequelae of electrical injuries. *Mononeuropathy* was the most frequent peripheral neuropathy we found, and was primarily seen in the arms or legs, probably due to these extremities often being the entry and/or exit points of the current. This is similar to what other studies with similar methodologies [4], and reviews [1, 2], have identified. Although all other peripheral nerve disorders, such as *polyneuropathy* and *disturbances of skin sensations*, were relatively rare in our population, our results indicated either an association with electrical injuries or a possible association. Thus, we were unable to disconfirm an association for any of our selected diagnoses related to the PNS. Together with the conclusions of previous research, the link between electrical injuries and peripheral nerve disorders seems to be well-established, and further research should focus on understanding the causal mechanisms, where different explanatory models are debated [2].

### Delayed onset

Several studies emphasise the possible delayed onset of neurological symptoms following electrical injuries, where months and years may pass after an electric shock, before the consequences become evident [2]. However, this is poorly documented [1]. We were able to investigate possible delayed onset for the seven most frequent diagnoses and found that the highest odds ratio was within the first six months of the injury for five of them, indicating that delayed onset is not the norm. But, only two of those diagnoses (Convulsions and Abnormal involuntary movements) did not show an increased risk in any time intervals after the first six months. Actually, for all but these two diagnoses we identified potential delayed onset of symptoms up until 5 years after the injury. However, as we use diagnoses as our outcome, we do not know whether this reflects delayed onset of symptoms or delayed diagnosing of the symptoms.

## Conclusion

Our results confirm that electrical injuries increase the risk of several neurological diseases and symptoms of the CNS or PNS in the years following the injury. Most often the diseases and symptoms are diagnosed within the first six months of the injury, but delayed onset of up to 5 years cannot be ruled out for some symptoms and diagnoses.

Some of the diagnoses were rare in our population, which limited our ability to identify associations, and this warrants cautious interpretation. Further studies are needed to confirm our findings, as are studies that examine the underlying mechanisms driving the associations.

The findings of this study may probably be generalised to other populations, especially in countries where access to healthcare is similar to Denmark’s, and where the habits and culture of diagnosing is similar.
